# Supplementary material for: Head-to-Head Comparison of BRAF/MEK Inhibitor Combinations Proposes Superiority of Encorafenib Plus Trametinib in Melanoma
Source: Cancers (Basel). 2022 Oct 8;14(19):4930. doi: 10.3390/cancers14194930 (PMC9564158; doi:10.3390/cancers14194930)
Supplement: Supplementary file 1 [file cancers-14-04930-s001.zip › cancers-1883595-supplementary.pdf]

Supplemental Material

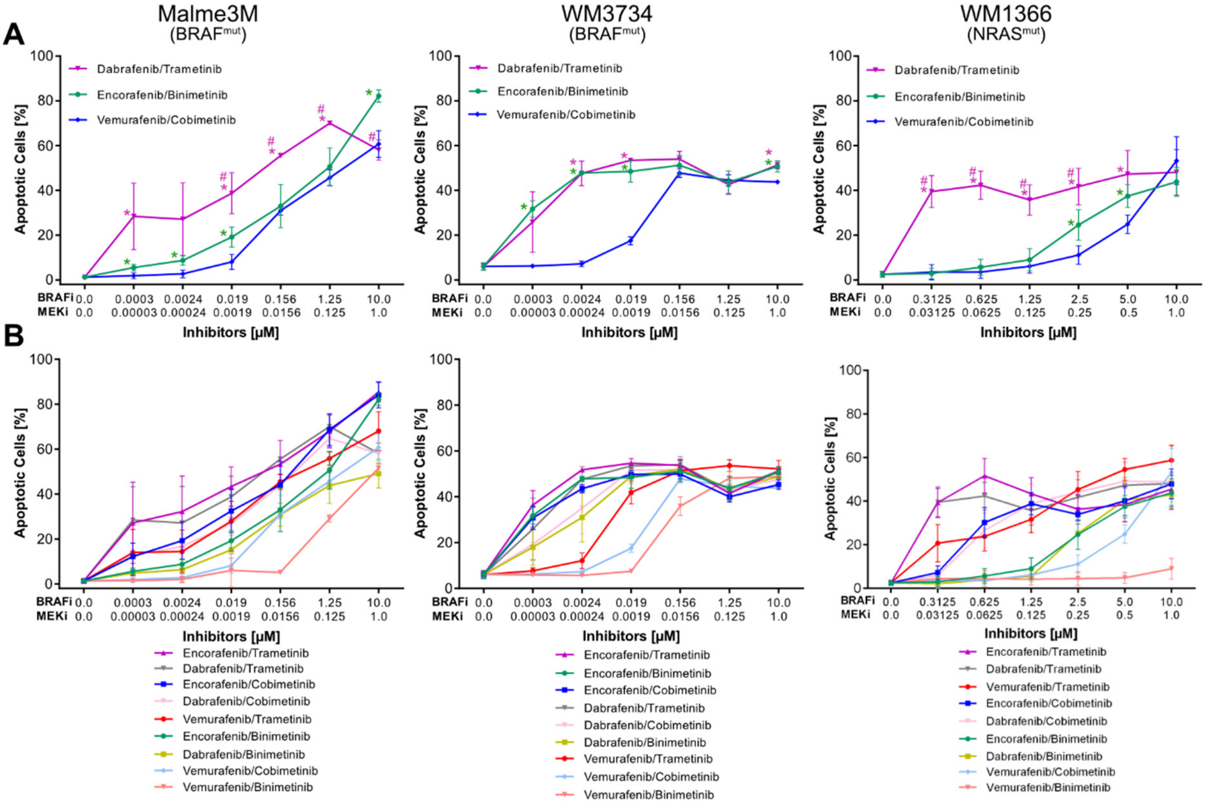

Figure S1: Encorafenib/trametinib represents the most efficient unconventional and dabrafenib/trametinib the best clinically approved inhibitor combination in BRAF^{mut} and NRAS^{mut} melanoma cells. Sub-G1 (apoptosis) of two BRAF^{mut} cell lines (Malme3M and WM3734) and one NRAS^{mut} cell line (WM1366) after treatment for 72 hours using different BRAFi/MEKi combinations with increasing concentrations of inhibitors (BRAF^{mut} 1:8 serial dilutions, NRAS^{mut} 1:2 serial dilutions). n ≥ 3, error bars indicate standard deviation. (A) Panel compares approved inhibitor combinations for BRAF^{mut} melanoma, * indicates significantly different values relative to vermurafenib/cobimetinib, # labels data points significantly different relative to encorafenib/binimetinib. Significance defined as $p<0.05$ based on two-tailed non-paired T-test. Table S3 shows complete list of p-values for all data points. (B) Panel summarizes all tested inhibitor combinations. Significances not shown.

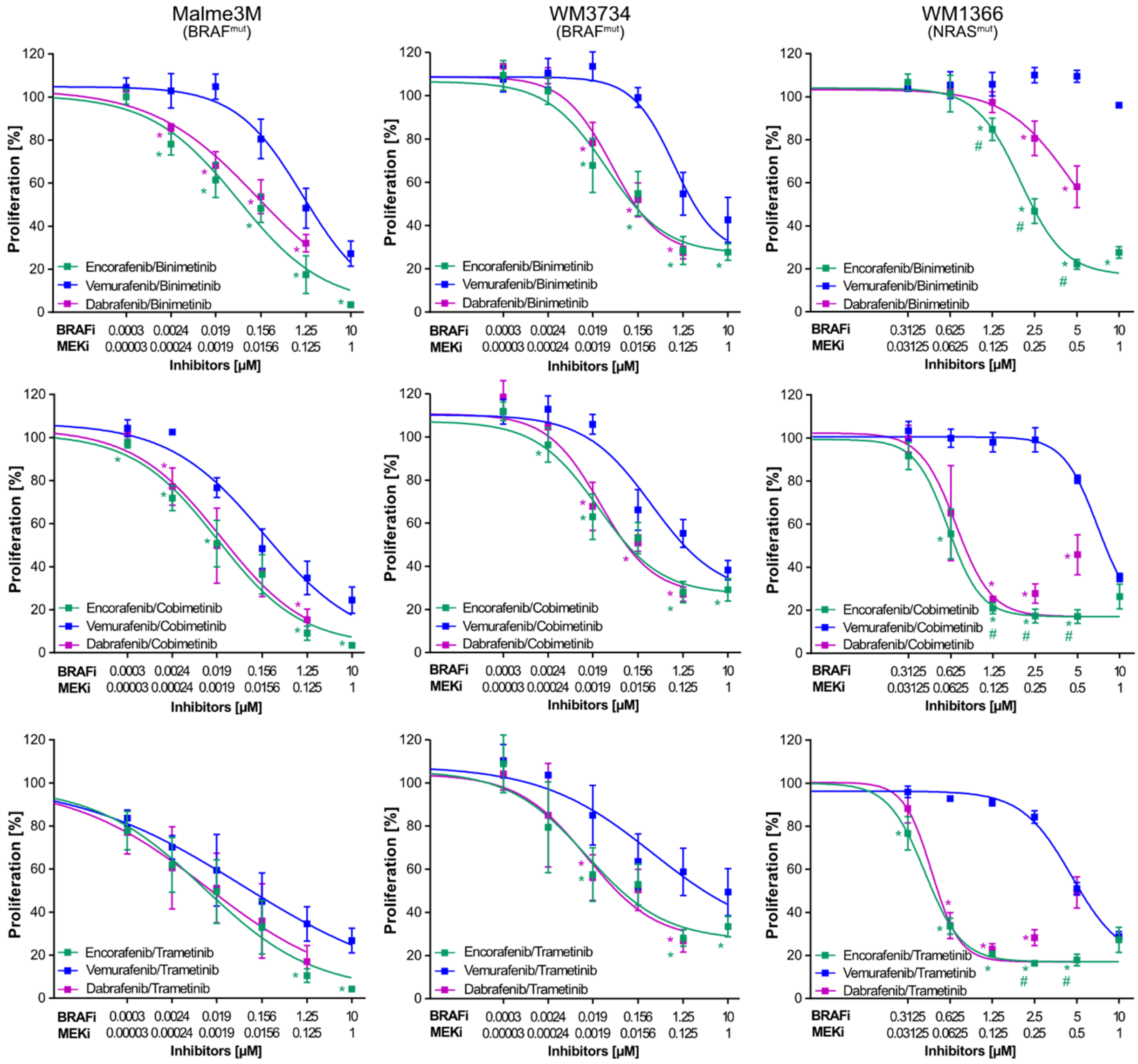

Figure S2: IC_{50} curves modeled from MUH proliferation data displaying differences for BRAFi. n ≥ 3, error bars indicate standard deviation, * indicates significantly different values relative to vermurafenib, # labels data points significantly different relative to dabrafenib. Significance defined as p<0.05 based on two-tailed non-paired T-test. Table S5 shows a complete list of p-values for all data points.

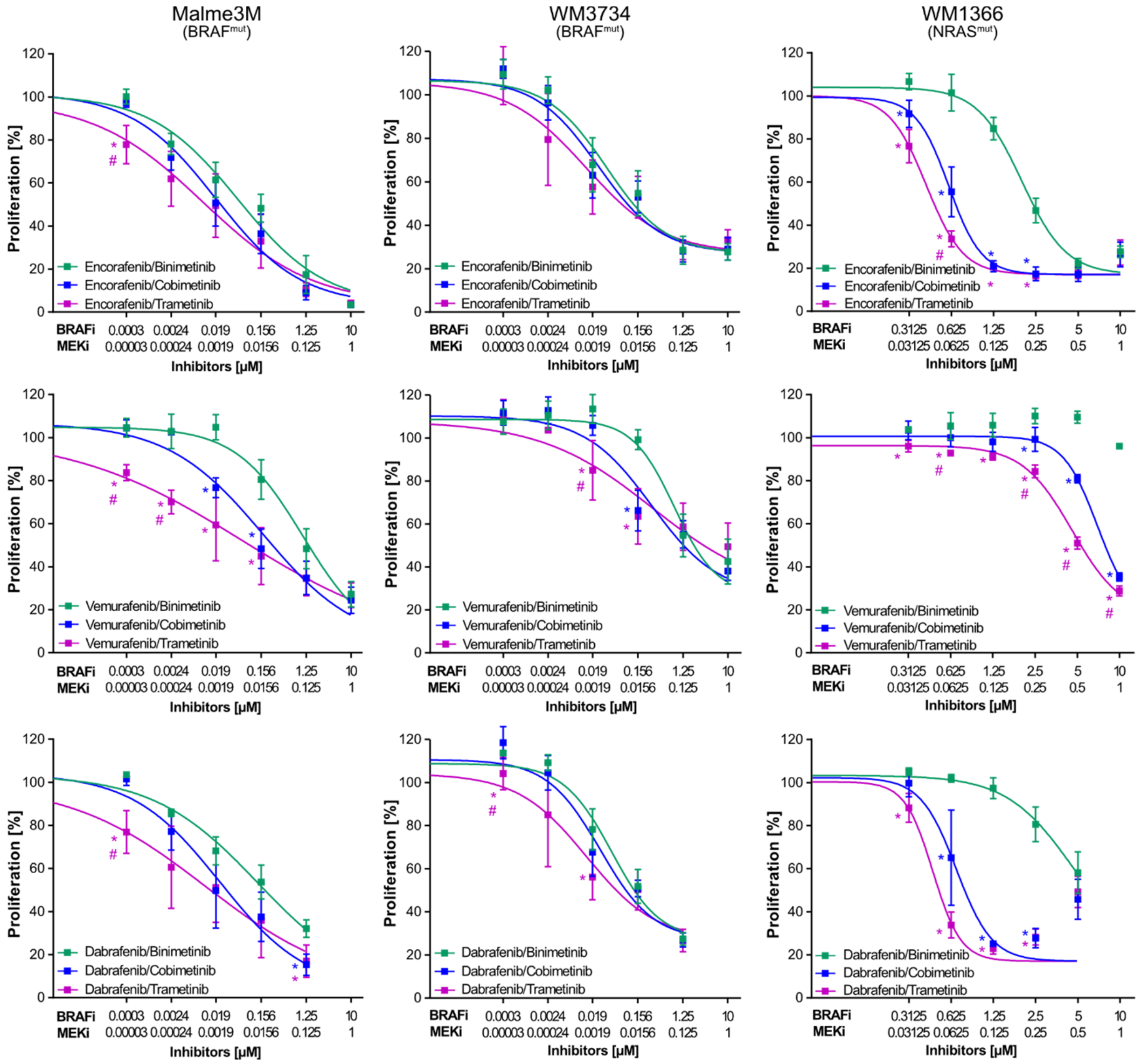

Figure S3: IC_{50} curves modeled from MUH proliferation data displaying differences for MEKi. $n \geq 3$, error bars indicate standard deviation, * indicates significantly different values relative to vermurafenib, # labels data points significantly different relative to dabrafenib. Significance defined as $p<0.05$ based on two-tailed non-paired T-test. Table S6 shows a complete list of p-values for all data points.

Table S1: List of p-values from subG1 analysis, related to Figure 1.

**Malme3M (BRAF^{mut})**									
BRAFi/MEKi [µM]	EB/VB	EB/DB	VB/DB	EC/VC	EC/DC	VC/DC	ET/VT	ET/DT	VT/DT
0.0003/0.00003	**0.00926**	0.50751	**0.00792**	**0.04350**	0.90822	**0.00362**	0.33336	0.92797	0.23650
0.0024/0.00024	**0.00996**	0.12183	**0.00708**	**0.02716**	0.67199	**0.01557**	0.17214	0.72018	0.30830
0.019/0.0019	0.05842	0.20106	0.10122	**0.01425**	0.42710	**0.00639**	0.10195	0.57198	0.21777
0.156/0.0156	**0.00774**	0.73956	**0.00103**	0.10076	0.97737	**0.01099**	0.28965	0.73124	**0.00756**
1.25/0.125	**0.01170**	0.36204	**0.03493**	**0.00759**	0.46498	**0.00498**	0.05507	0.64993	**0.00133**
10/1	**0.00004**	**0.00117**	0.53419	**0.00816**	**0.01218**	0.67732	**0.03543**	**0.00172**	0.15356

**WM3734 (BRAF^{mut})**									
BRAFi/MEKi [µM]	EB/VB	EB/DB	VB/DB	EC/VC	EC/DC	VC/DC	ET/VT	ET/DT	VT/DT
0.0003/0.00003	**0.00029**	0.05337	0.05807	**0.00115**	0.19782	0.13304	**0.00151**	0.29019	0.08136
0.0024/0.00024	**0.00000**	0.05399	**0.01496**	**0.00001**	0.26774	**0.01339**	**0.00004**	0.26745	**0.00068**
0.019/0.0019	**0.00012**	0.89402	**0.00002**	**0.00002**	0.33596	**0.00004**	**0.01561**	0.40526	**0.01722**
0.156/0.0156	**0.01223**	0.74520	**0.00311**	0.28001	0.56392	0.26384	0.50961	0.90949	0.49747
1.25/0.125	0.24995	0.97658	0.09721	0.08291	0.15193	0.50204	**0.00478**	0.71378	**0.01994**
10/1	0.51134	0.24367	0.95457	0.30740	0.30355	0.06184	0.68383	0.84418	0.77401

**WM1366 (NRAS^{mut})**									
BRAFi/MEKi [µM]	EB/VB	EB/DB	VB/DB	EC/VC	EC/DC	VC/DC	ET/VT	ET/DT	VT/DT
0.3125/0.03125	0.48307	0.61299	0.26616	0.14906	0.47553	0.28519	**0.01365**	0.96846	**0.01501**
0.625/0.0625	0.62789	0.46298	0.83763	**0.00038**	0.51007	**0.00175**	**0.00179**	0.11670	**0.00719**
1.25/0.125	0.13613	0.23894	0.50098	**0.00003**	0.99626	**0.00044**	0.05027	0.17498	0.40851
2.5/0.25	**0.00177**	0.90909	**0.00213**	**0.00011**	**0.04996**	**0.00031**	0.14343	0.34469	0.56205
5/0.5	**0.00003**	0.61931	**0.00053**	**0.00867**	0.14892	**0.00222**	**0.01339**	0.22404	0.26091
10/1	**0.00013**	0.84124	**0.00014**	0.42749	0.84743	0.56873	0.06183	0.71664	0.13577

E…encorafenib; V…vemurafenib; D…dabrafenib; B…binimetinib; C…cobimetinib; T…trametinib; bold numbers indicate significant values with $p<0.05$ as determined by two-tailed non-paired T-test.

Table S2: List of p-values from subGl analysis, related to Figure 2.

				**Malme3M (BRAF^{mut})**					
BRAFi/MEKi [µM]	EB/EC	EB/ET	EC/ET	VB/VC	VB/VT	VC/VT	DB/DC	DB/DT	DC/DT
0.0003/0.00003	0.13393	0.10823	0.24608	0.56391	0.10224	0.11435	**0.00993**	0.05117	0.14475
0.0024/0.00024	0.09811	0.06342	0.27715	0.60258	0.09217	0.10817	**0.03495**	0.09032	0.34912
0.019/0.0019	0.09649	**0.01367**	0.22261	0.68431	**0.03099**	**0.02283**	**0.01850**	**0.01148**	0.12348
0.156/0.0156	0.25303	0.07165	0.34600	**0.00002**	**0.00003**	**0.00289**	**0.03055**	**0.00123**	**0.01289**
1.25/0.125	**0.04594**	0.05294	0.90242	**0.00193**	**0.00016**	**0.01943**	**0.01694**	**0.00477**	0.12677
10/1	0.62594	0.34050	0.78321	0.05494	**0.02942**	0.29196	0.22047	0.11662	0.98670

				**WM3734 (BRAF^{mut})**					
BRAFi/MEKi [µM]	EB/EC	EB/ET	EC/ET	VB/VC	VB/VT	VC/VT	DB/DC	DB/DT	DC/DT
0.0003/0.00003	0.80307	0.33424	0.29694	0.51316	0.09424	0.16057	0.88814	0.43371	0.55842
0.0024/0.00024	**0.02353**	**0.01095**	**0.00217**	0.11515	**0.03106**	0.07854	0.67705	0.07498	0.15781
0.019/0.0019	0.69042	0.11063	**0.03500**	**0.00066**	**0.00030**	**0.00141**	0.29951	0.06642	0.24514
0.156/0.0156	0.66666	0.45381	0.11214	**0.01059**	**0.01330**	0.29782	0.91802	0.46549	0.57790
1.25/0.125	0.30807	0.53416	0.50663	0.17565	0.06851	**0.01232**	0.82298	0.75291	0.85798
10/1	**0.04460**	0.81549	**0.01538**	0.08502	0.34559	**0.01959**	0.41249	0.07043	0.08010

				**WM1366 (NRAS^{mut})**					
BRAFi/MEKi [µM]	EB/EC	EB/ET	EC/ET	VB/VC	VB/VT	VC/VT	DB/DC	DB/DT	DC/DT
0.3125/0.03125	0.06662	**0.00005**	**0.00011**	0.69475	**0.01225**	**0.00967**	**0.04172**	**0.00006**	**0.00010**
0.625/0.0625	**0.00073**	**0.00004**	**0.00661**	0.74687	**0.00240**	**0.00148**	**0.00197**	**0.00004**	**0.02175**
1.25/0.125	**0.00016**	**0.00024**	0.34235	0.38142	**0.00019**	**0.00032**	**0.00035**	**0.00015**	0.59852
2.5/0.25	**0.04686**	0.05199	0.54440	**0.04445**	**0.00009**	**0.00032**	**0.01270**	**0.02427**	0.68364
5/0.5	0.55919	0.85258	0.74865	**0.00017**	**0.00000**	**0.00011**	0.24622	0.39733	0.78858
10/1	0.42509	0.78017	0.69431	**0.00028**	**0.00002**	0.42301	0.31539	0.41892	0.90006

E…encorafenib; V…vemurafenib; D…dabrafenib; B…binimetinib; C…cobimetinib; T…trametinib; bold numbers indicate significant values with $p<0.05$ as determined by two-tailed non-paired T-test.

Table S3: List of p-values from subGl analysis, related to Figure S1A.

	**Malme3M (BRAF^{mut})**			**WM3734 (BRAF^{mut})**			**WM1366 (NRAS^{mut})**			
BRAFi/MEKi [µM]	EB/VB	EB/DB	VB/DB	EB/VB	EB/DB	VB/DB	BRAFi/MEKi [µM]	EB/VB	EB/DB	VB/DB
0.0003/0.00003	**0.03246**	0.05644	**0.03679**	**0.00030**	0.50912	0.06629	0.3125/0.03125	0.48307	0.61299	0.26616
0.0024/0.00024	**0.01939**	0.12323	0.06077	**0.00000**	0.96149	**0.00024**	0.625/0.0625	0.62789	0.46298	0.83763
0.019/0.0019	**0.02677**	**0.02979**	**0.00562**	**0.00047**	0.14939	**0.00001**	1.25/0.125	0.13613	0.23894	0.50098
0.156/0.0156	0.73636	**0.01638**	**0.00004**	0.29814	0.44901	0.05649	2.5/0.25	**0.00177**	0.90909	**0.00213**
1.25/0.125	0.40243	**0.01598**	**0.00035**	0.80294	0.79512	0.54429	5/0.5	**0.00003**	0.61931	**0.00053**
10/1	**0.00471**	**0.00151**	0.58050	**0.00990**	0.72283	**0.00262**	10/1	**0.00013**	0.84124	**0.00014**

E…encorafenib; V…vemurafenib; D…dabrafenib; B…binimetinib; C…cobimetinib; T…trametinib; bold numbers indicate significant values with $p<0.05$ as determined by two-tailed non-paired T-test.

Table S4: Comparison of IC_{50} values for single and combined inhibitor treatment.

Malme3M (BRAF^mut)				
**IC_{50} (µM)**	**Single BRAFi**	**Binimetinib**	**Cobimetinib**	**Trametinib**
**Single MEKi**		2.12400	0.08621	0.00922
**Encorafenib**	0.0841	0.0537	0.0194	0.0107
		0.00537	0.00194	0.00107
**Vemurafenib**	3.0400	1.0780	0.1684	0.0620
		0.10780	0.01684	0.00620
**Dabrafenib**	0.3286	0.1294	0.0227	0.0119
		0.01294	0.00227	0.00119

WM3734 (BRAF^mut)				
**IC_{50} (µM)**	**Single BRAFi**	**Binimetinib**	**Cobimetinib**	**Trametinib**
**Single MEKi**		3.75800	0.48130	0.10690
**Encorafenib**	0.0466	0.0356	0.0247	0.0143
		0.00356	0.00247	0.00143
**Vemurafenib**	2.6450	0.8345	0.2885	0.3022
		0.08345	0.02885	0.03022
**Dabrafenib**	0.0546	0.0458	0.0271	0.0145
		0.00458	0.00271	0.00145

WM1366 (NRAS^mut)				
**IC_{50} (µM)**	**Single BRAFi**	**Binimetinib**	**Cobimetinib**	**Trametinib**
**Single MEKi**		NC	12.08000	4.41400
**Encorafenib**	NC	1.9830	0.5985	0.4139
		0.19830	0.05985	0.04139
**Vemurafenib**	NC	NC	6.9750	4.6110
		NC	0.69750	0.46110
**Dabrafenib**	NC	4.6610	0.6812	0.4631
		0.46610	0.06812	0.04631

IC_{50}…half maximum inhibitory concentration, NC…not converged

Table S5: List of p-values from MUH proliferation data, related to Figure S2.

Malme3M (BRAF^{mut})									
BRAFi/MEKi [µM]	EB/VB	EB/DB	VB/DB	EC/VC	EC/DC	VC/DC	ET/VT	ET/DT	VT/DT
0.0003/0.00003	0.23484	0.18011	0.69762	**0.04834**	0.10860	0.38831	0.34917	0.90818	0.32922
0.0024/0.00024	**0.01038**	0.06477	**0.02239**	**0.00090**	0.42776	**0.00739**	0.36330	0.92152	0.44996
0.019/0.0019	**0.00170**	0.31932	**0.00197**	**0.01879**	0.93199	0.06008	0.48216	0.90218	0.57006
0.156/0.0156	**0.00768**	0.40761	**0.01865**	0.18787	0.90605	0.27080	0.31847	0.82795	0.51007
1.25/0.125	**0.01406**	0.06191	**0.04905**	**0.00614**	0.14929	**0.02160**	**0.00876**	0.23519	0.05049
10/1	**0.00227**	ND	ND	**0.00405**	ND	ND	**0.00251**	ND	ND

WM3734 (BRAF^{mut})									
BRAFi/MEKi [µM]	EB/VB	EB/DB	VB/DB	EC/VC	EC/DC	VC/DC	ET/VT	ET/DT	VT/DT
0.0003/0.00003	0.70308	0.26181	0.08809	0.94424	0.18218	0.20030	0.88113	0.57058	0.35288
0.0024/0.00024	0.11532	0.09949	0.74236	**0.01767**	0.19744	0.15623	0.11083	0.74191	0.24507
0.019/0.0019	**0.00066**	0.23365	**0.00093**	**0.00029**	0.55804	**0.00075**	**0.04004**	0.86969	**0.02539**
0.156/0.0156	**0.00022**	0.67198	**0.00005**	0.07104	0.60394	**0.02388**	0.25891	0.72112	0.17495
1.25/0.125	**0.00445**	0.77767	**0.00189**	**0.00056**	0.73079	**0.00024**	**0.00318**	0.68895	**0.00343**
10/1	**0.03813**	ND	ND	**0.03774**	ND	ND	**0.04398**	ND	ND

WM1366 (NRAS^{mut})									
BRAFi/MEKi [µM]	EB/VB	EB/DB	VB/DB	EC/VC	EC/DC	VC/DC	ET/VT	ET/DT	VT/DT
0.3125/0.03125	0.27420	0.56233	0.28905	0.05770	0.19300	0.45232	**0.01494**	0.12138	0.13772
0.625/0.0625	0.55579	0.92364	0.41584	**0.00342**	0.53766	0.05522	**0.00001**	0.94300	**0.00007**
1.25/0.125	**0.00903**	**0.03923**	0.12049	**0.00001**	**0.04340**	**0.00001**	**0.00000**	0.24061	**0.00000**
2.5/0.25	**0.00009**	**0.00397**	**0.00442**	**0.00003**	**0.02914**	**0.00007**	**0.00000**	**0.00639**	**0.00003**
5/0.5	**0.00000**	**0.00340**	**0.00094**	**0.00001**	**0.00713**	**0.00315**	**0.00012**	**0.00219**	0.69943
10/1	**0.00000**	ND	ND	0.06481	ND	ND	0.69309	ND	ND

E…encorafenib; V…vemurafenib; D…dabrafenib; B…binimetinib; C…cobimetinib; T…trametinib; ND…not determined; bold numbers indicate significant values with $p<0.05$ as determined by two-tailed non-paired T-test.

Table S6: List of p-values from MUH proliferation data, related to Figure S3.

Malme3M (BRAF^{mut})									
BRAFi/MEKi [µM]	EB/EC	EB/ET	EC/ET	VB/VC	VB/VT	VC/VT	DB/DC	DB/DT	DC/DT
0.0003/0.00003	0.29511	**0.01589**	**0.02120**	0.95532	**0.00324**	**0.00272**	0.36441	**0.01017**	**0.01504**
0.0024/0.00024	0.23360	0.10863	0.28399	0.94449	**0.00436**	**0.00060**	0.16801	0.08452	0.23952
0.019/0.0019	0.24524	0.28623	0.90821	**0.00289**	**0.01144**	0.15881	0.15961	0.16493	0.92187
0.156/0.0156	0.14502	0.13535	0.71754	**0.01269**	**0.01847**	0.73149	0.11439	0.18000	0.89552
1.25/0.125	0.19667	0.26341	0.63154	0.12361	0.12402	0.97069	**0.01111**	**0.03714**	0.76095
10/1	0.91088	0.08211	0.05383	0.60273	0.91426	0.66938	ND	ND	ND

WM3734 (BRAF^{mut})									
BRAFi/MEKi [µM]	EB/EC	EB/ET	EC/ET	VB/VC	VB/VT	VC/VT	DB/DC	DB/DT	DC/DT
0.0003/0.00003	0.55416	0.93331	0.66520	0.35930	0.64264	0.77152	0.25710	**0.04641**	**0.03625**
0.0024/0.00024	0.29233	0.08428	0.18482	0.63917	0.14101	0.05638	0.33315	0.09244	0.17180
0.019/0.0019	0.57584	0.28861	0.52877	0.10934	**0.01454**	0.03417	0.20502	**0.02109**	0.18266
0.156/0.0156	0.80420	0.80173	0.97327	**0.00077**	**0.00331**	0.76684	0.82688	0.81828	0.92817
1.25/0.125	0.93005	0.92718	0.99647	0.92636	0.62501	0.60976	0.85754	0.82579	0.93268
10/1	0.69534	0.11824	0.27196	0.46664	0.44221	0.11655	ND	ND	ND

WM1366 (NRAS^{mut})									
BRAFi/MEKi [µM]	EB/EC	EB/ET	EC/ET	VB/VC	VB/VT	VC/VT	DB/DC	DB/DT	DC/DT
0.3125/0.03125	**0.02483**	**0.00383**	0.05993	0.89550	**0.00929**	0.06674	0.22332	**0.01353**	0.09701
0.625/0.0625	**0.00536**	**0.00024**	**0.03684**	0.28948	**0.02607**	**0.04390**	**0.04536**	**0.00005**	0.07769
1.25/0.125	**0.00005**	**0.00003**	0.90252	0.13312	**0.01192**	0.06563	**0.00001**	**0.00002**	0.19692
2.5/0.25	**0.00146**	**0.00084**	0.65062	**0.04903**	**0.00069**	**0.01513**	**0.00056**	**0.00050**	0.91116
5/0.5	0.08528	0.10404	0.74835	**0.00015**	**0.00001**	**0.00011**	0.18918	0.26927	0.65211
10/1	0.75555	0.92560	0.86538	**0.00000**	**0.00000**	**0.01922**	ND	ND	ND

E…encorafenib; V…vemurafenib; D…dabrafenib; B…binimetinib; C…cobimetinib; T…trametinib; ND…not determined; bold numbers indicate significant values with $p<0.05$ as determined by two-tailed non-paired T-test.
